# Influence of biodegradable polymer membrane on new bone formation and biodegradation of biphasic bone substitutes: an animal mandibular defect model study

**DOI:** 10.1186/s40902-020-00280-5

**Published:** 2020-10-15

**Authors:** Jeong-Kui Ku, Young-Kyun Kim, Pil-Young Yun

**Affiliations:** 1 Department of Oral and Maxillofacial Surgery, Section of Dentistry, Section of Dentistry, Armed Forces Capital Dental Hospital, Armed Forces Medical Command, 81, Saemaul-ro 117, Seongnam, 13634 Bundang-gu Korea; 2 Department of Oral and Maxillofacial Surgery, Section of Dentistry, Seoul National University Bundang Hospital, 300 Gumi-dong, Bundang-gu, Gyeonggi-do 463-707 Korea; 3 Department of Dentistry and Dental Research Institute, School of Dentistry, Seoul National University, 101 Daehak-ro, Seoul, 03080 Jongno-gu Korea

**Keywords:** Beta-tricalcium phosphate, Biphasic calcium phosphate, Bone regeneration, Hydroxyapatite, Membrane, Polycaprolactone

## Abstract

**Purpose:**

The purpose of this study was to evaluate the influence of biodegradable polycaprolactone membrane on new bone formation and the biodegradation of biphasic alloplastic bone substitutes using animal models.

**Materials and methods:**

In this study, bony defect was formed at the canine mandible of 8 mm in diameter, and the defects were filled with Osteon II. The experimental groups were covered with Osteoguide as barrier membrane, and the control groups were closed without membrane coverage. The proportion of new bone and residual bone graft material was measured histologically and histomorphometrically at postoperative 4 and 8 weeks.

**Results:**

At 4 weeks, the new bone proportion was similar between the groups. The proportion of remaining graft volume was 27.58 ± 6.26 and 20.01 ± 4.68% on control and experimental groups, respectively (*P* < 0.05). There was no significant difference between the two groups in new bone formation and the amount of residual bone graft material at 8 weeks.

**Conclusion:**

The biopolymer membrane contributes to early biodegradation of biphasic bone substitutes in the jaw defect but it does not affect the bone formation capacity of the bone graft.

## Background

Cystic lesion is frequently developed in the jaw, which has higher risk of trauma and infection compared to other bones [1]. The surgical enucleation of cyst is the standard protocol unless an anatomical limitations [1, 2]. In general, the enucleation includes bony window formation to access the cystic lesion, resulting a large postoperative defect. Healing of the defect is affected by carious conditions such as size of the defect, condition of periosteum, soft tissue covering, and general conditions of patient [3]. Without bone graft, however, the spontaneous bone regeneration was not completed within 6 months [4]. Many research reported that bone graft could reduce the bone-healing period and the risk of pathologic fracture after the enucleation [4–7]. In addition, Forssell et al. reported higher recurrence rate was in the larger size of cyst (≥ 4 cm) [8]. In order to detect the recurrence, early increase in bone density is important to be continuously observed without change in volume and density after the enucleation [9, 10].

Although the gold standard for bone graft is still autogenous bone, alloplastic bone has been developed in various ways for alternative option. Osteon II (Genoss Co., Suwon, Korea) altered to a reinforced microstructure with small pore size of < 250 μm and inter-connective porosity from Osteon I (Genoss Co., Suwon, Korea), a hydroxyapatite (HA) scaffold with pore size of 300-500 μm [11]. The Osteon II is classified as a biphasic scaffold with combination of HA (30%) and resorbable ß-tricalcium phosphate (ß-TCP, 70%). Because of the composition of ß-TCP, this biphasic scaffold showed a degradable aspect and could be anticipated to new bone formation as much as the resorption [11]. The characteristic of micro- and macro-porosity structure is also advantageous for the attachment and proliferation of osteoblasts [12].

The effectiveness of using absorbable membrane has been controversial to enhance new bone formation in the cystic defect. Some studies suggested that the membrane could be necessary to additional stabilization process, especially, in a large cystic defect ≥ 3 cm and loss of periosteum or bone wall [13, 14]. On the other hand, Santamaria et al. showed that bone graft using membranes does not contribute to increase bone regeneration [15]. Therefore, the efficacy of a membrane has still been controversial even in the large cystic defect [5–7]. Osteoguide (Genoss Co., Suwon, Korea) is 5-50 μm microporous membrane with 0.13 mm in thickness, and it is manufactured by polycaprolactone (PCL), which is a biodegradable polymer with excellent mechanical properties, and cell proliferation abilities [16, 17]. The purpose of this experiment was to evaluate the effectiveness of resorbable PCL membrane for new bone formation and biodegradation of the biphasic alloplastic bone substitute in canine mandibular defect. We hypothesized that the membrane, covering the bone substitute, could contribute to enhance bone regeneration capacity and early bone formation.

## Materials and methods

Guidelines regarding the care of animal research subjects were strictly followed the ARRIVE guidelines and approved by the Institutional Animal Care and Use Committee of Seoul National University Bundang Hospital, Korea (IACUC No. BA1205-104/034-01). Researches support that canines show more promise as animal models for the testing of bone implant materials because their bone structures had more similarities to the humans. Beagle was selected as animal models.

### Animal preparation and surgical procedure

Four beagle dogs (5-6 months old, weighing 8-10 kg in good health) were used in the study. Beagles were fed with commercial diet (Dog Chow GoldPet, #35520, Cargill Agri Purina, Inc., Pyungtaek, Korea) and housed in individual cages. The animals were prepared with a subcutaneous injection of atropine 0.005 mg/kg (Daihan Pharm. Co., Ansan, Korea) under supine position and anesthetized with an intramuscular injection of zoletil (Zoletil50, Virbac S.A., Carros, France) 5.0 mg/kg and xylazine (Rompun, Bayer Korea, Ansan, Korea) 0.2 mg/kg after 15 min. After endotracheal intubation of a 6.5-sized tube, general anesthesia was maintained with enflurane 2.2% (JW Pharmaceutical, Hwasung, Korea) and oxygen level at 3.0 l/min. Animals were injected intramuscularly with cefazolin 30 mg/kg (Chongkundang Pharm, Cheonan, Korea) before surgical procedures.

**Fig. 1:**
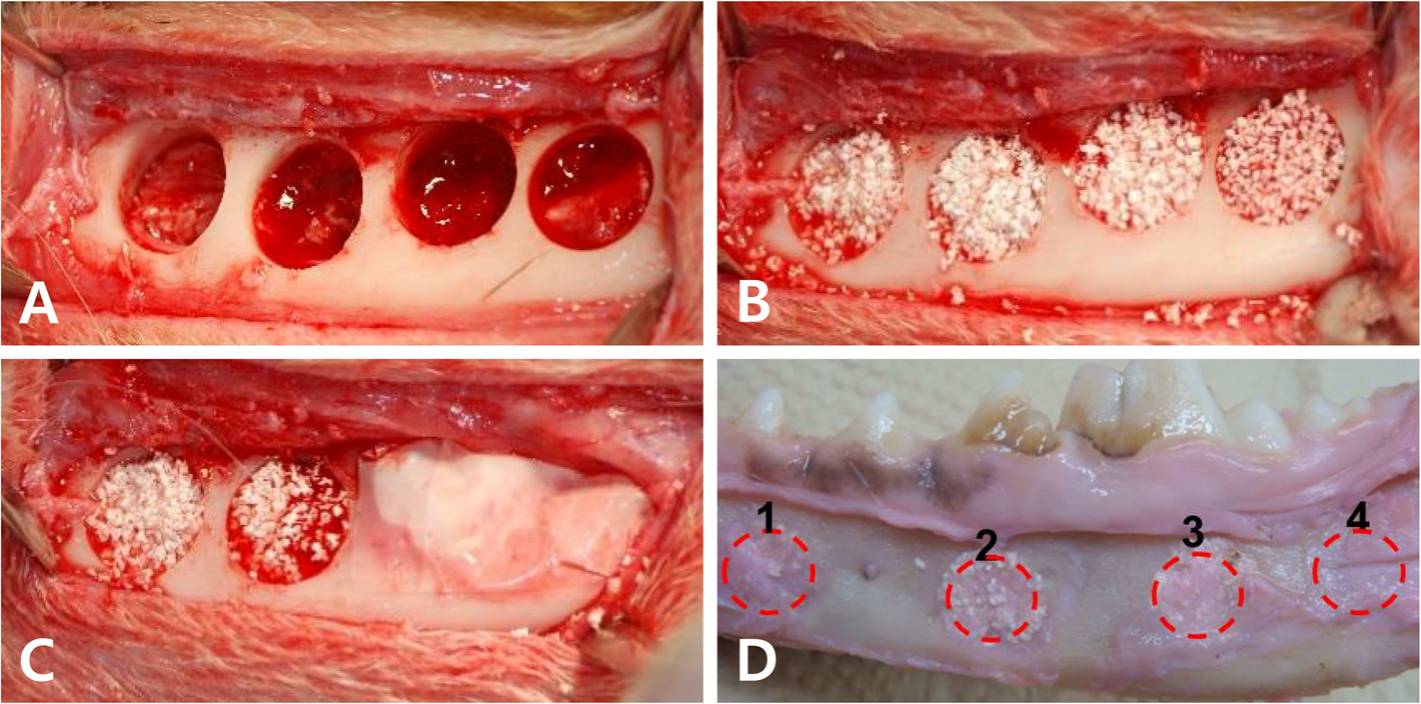
Experimental photograph. **a** Drilling holes of 8.0 mm were made on mandible of dog. **b** The defects were filled with OSTEON II (Genoss Co., Suwon, Korea). **c** Two of four holes were covered with Osteoguide membrane (Genoss Co., Suwon, Korea). **d** Sacrifice was performed at 4 and 8 weeks after surgery

The surgical field was scrubbed with povidone-iodine solution. Local anesthesia for hemostasis using 2% lidocaine with 1:100,000 epinephrine (Yuhan Co, Ltd., Seoul, Korea) was injected on both submandibular areas. Skin incisions were made on submandibular area, and subperiosteal dissection was raised to expose lateral surface of mandibular body area. A standardized 4 round bone defects of 8.0 mm size simulating cystic lesion with bony window using large trephine bur on both mandible [18]. Each depth of defect was made until the lateral cortex was completely removed. Cold saline irrigation was performed to prevent heating by the burr (Fig. 1a)

Each bony defects was filled with OSTEON II (HA:β-TCP = 3:7, Genoss Co., Suwon, Korea) The experimental defects were covered Osteoguide membrane, and the others were closed without membrane coverage (Fig. 1b and c). All surgical sites underwent primary closure using polyglactin 4-0 (Vicryl, Ethicon, Menlo Park, CA). Post-operatively, 1.0 ml of dexamethasone-21-isonicotinate (Voren, Boehringer Ingelheim Korea Ltd., Seoul, Korea) was injected for once, and clemizole penicillin G and sodium penicillin G (Antipen-SM, WooGene B&G Ltd., Seoul, Korea) 1.0 ml/10.0 kg were injected for three times on every third day. Two beagles were sacrificed each time on 4 and 8 weeks later through formalin perfusion (Fig. 1d).

### Histologic analysis

Block sections (8 × 8 × 5 mm) including grafted sites were harvested. The sections were fixed with 10% buffered neutral formalin (Sigma Aldrich Co. LLC., St. Louis, USA) for 2 weeks and decalcified in formic acid (Shadon TBD-1, Thermo Fisher Scientific Inc., Kalamazoo, USA) following water rinse. The specimens were decalcified in tissue processor (Shadon Citadel 2000, Thermo Fisher Scientific Inc., Kalamazoo, USA) and embedded in paraffin with embedding center (Shadon Histocentre 3, Thermo Fisher Scientific Inc., Kalamazoo, USA). Serial sections of 3.0 μm in thickness were cut using Microtome (Shadon Finesse 325, Thermo Fisher Scientific Inc., Kalamazoo, USA). And each specimen was stained with hematoxylin and eosin (H&E).

And dehydration of the specimens was done in ethanol. Specimens were embedded with ethanol mixed resin solution (Technovit 7200 VLC, Heraeus Kulzer, Hanau, Germany). Blocks were fixed in light polymerization unit (EXAKT 520, EXAKT Technologies Inc., Oklahoma City, USA) and sections of 300 μm thickness were cut using EXAKT cutting system (EXAKT 300 CP, EXAKT Technologies Inc., Oklahoma City, USA) and grinded in 35 μm thickness. Each specimen was stained with H&E and Goldner-Trichrome stains.

To obtain histomorphometric measurements, image was acquired from a light microscope (BX51, Olympus Co., Tokyo, Japan) connected with computer, CCD camera (SPOT Insight 2Mp scientific CCD digital camera system, Diagnostic Instruments Inc., Sterling Heights, USA), and adaptor (U-CMA3, Olympus Co., Tokyo, Japan). SPOT Software V4.0 (Diagnostic Instruments Inc., USA) was used for the image analysis. Tissue sample images were taken at low magnifications for histologic examination (× 12.5) and histomorphometric measurement (× 40). New bone formation was measured using Pro Plus® (Media Cybernetics Inc., Warrendale, USA) analysis software.

**Fig. 2:**
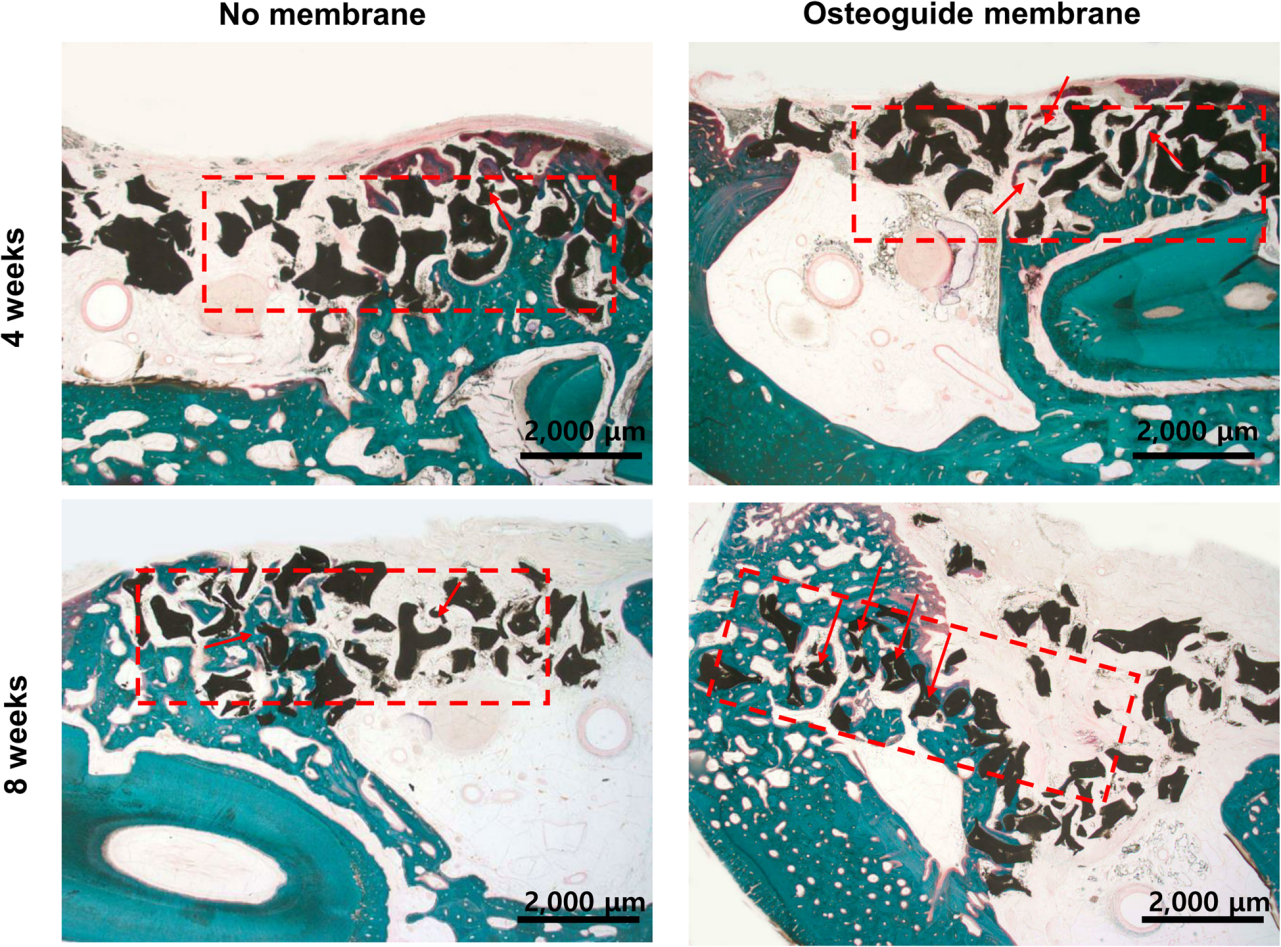
Histologic findings on the experimental and control groups at 4 and 8 weeks postoperatively. Newly formed bone (green) and remaining graft (black) volumes were calculated based on the total area (12 mm^2^ area, red square), respectively. The biodegradation in osteon was pointed as arrow. At 4 weeks, immature new bone was formed around graft material, large amount of woven bone with small portion of mature bone was formed around graft material. At 8 weeks, large amount of new bone was formed around graft material, and trabecular containing many osteoblasts were detected around graft material. The Osteoguide showed resorption progression and calcium particles are observed on the Osteoguide area without inflammation tissue. The covered shape of osteoguide was irregular pattern. There was shown to delayed new bone formation as fibrous tissue formed earlier than osteogenesis

For histometric evaluation in this experiment, because OSTEON II was distributed irregularly in the entire treatment area, it is impossible to accurately evaluate the area of the treatment site, which is a conventional measurement method. Newly formed bony volume (BV) was evaluated within 12 mm^2^. The boundary between the defect and the normal bone is first set, and the area of the newly formed bone is calculated as the percentage of the total area of the defect. Remaining grafting volume (RG) was evaluated as same as BV (Fig. 2).

In order to compare the differences in the degree of osteogenesis according to the presence or absence of membrane in the control and experimental groups, the Mann-Whitney test statistical analysis was performed at the significant probability of 95% using statistic program SPSS, version 25.0 (SPSS Inc., Chicago, IL, USA).

## Results

There were no any complications such as inflammatory reaction, wound dehiscence and infection. Histologic results were shown that the fibrous tissue was formed earlier than osteogenesis. Osteoguide was showed resorption progression, and calcium particles were observed on Osteoguide area without inflammation tissue. The covered shape of Osteoguide was irregular pattern. At 4 weeks, immature new bone was formed around the graft materials. The large amount woven bone was formed around the grafts with small portion of mature bone. At 8 weeks, a large amount of new bone was formed around graft material. There was shown a relatively large ratio of woven/lamellar bone, and trabecular bone was detected around graft material with containing many osteoblasts (Fig. 2).

**Fig. 3:**
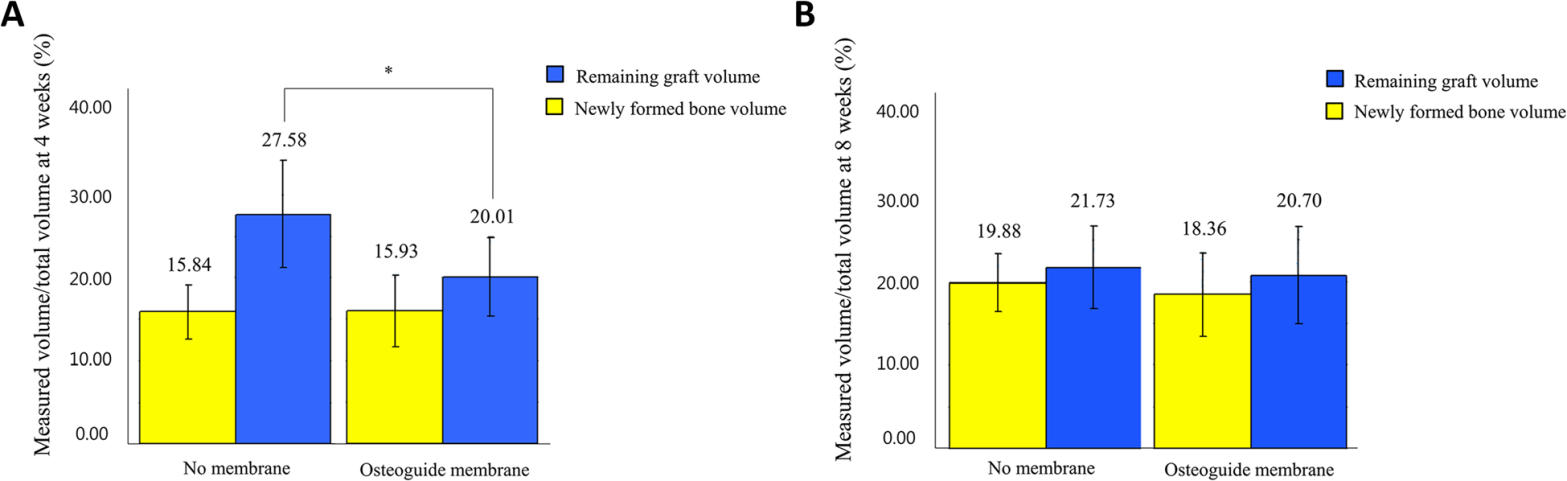
Histologic analysis on the experimental and control groups. **a** At 4 weeks, newly formed bone volume was similar between the groups, but remaining graft volume was higher in no membrane group than osteoguide membrane group. **b** At 8 weeks, there was no significantly difference of newly formed bone and remaining graft volumes between the groups. * Krustal-Wallis Test is significant at the 0.05 level (2-tailed)

At 4 weeks, the newly formed bone volume was 15.84 ± 3.43 and 15.93 ± 4.31% on the control and experimental groups, respectively. The remaining graft volume was 27.58 ± 6.26 and 20.01 ± 4.68% on the control and experimental groups, respectively, with statistically difference (*P* < 0.05, Fig. 3a). At 8 weeks, the newly formed bone and the remaining graft volume were 19.88 ± 3.43 and 18.36 ± 5.00% on the control group and 21.73 ± 5.01 and 20.70 ± 6.05% on experimental groups, respectively (Fig. 3b).

## Discussion

Although the proportion of new bone was not different, the proportion of remaining grafts was reduced with covering the absorbable membrane in early stage (at 4 weeks in canine). Considering that the 20% of the bone substitutes remained similarly even after 8 weeks, the membrane was seemed to have contribute to the rapid biodegradation of ß-TCP by stabilization of the graft, which could demonstrate both immobilization of osteons and early formed blood clots.

Several studies showed effectiveness of autogenous and alloplastic bone substitutes in the cystic defect [5–7]. In general, alloplastic material has excellent mechanical properties including volume stability against compression of soft tissue [12, 19]. Osteon II, using in this study, has ratio of HA:β-TCP as 3:7, and similar composition to human bones [20]. In particular, the pattern of bone regeneration with biodegradation of *β*-TCP was similar to intramembranous ossification, which is the way of jaw bone formation [21, 22]. In recent animal study, *there* reported a low rate of ossification regardless of *β*-TCP alone or when mixed with autogenous bone or HA, but similar healing patterns compared to autogenous bone graft [23–25]. Further, Horch et al. [26] showed complete bone regeneration with *β*-TCP alone after cyst enucleation at postoperative 12 month. These studies collaborated with our study, which was observed stable new bone formation with maintained 20% of graft. Therefore, the alloplastic bone substitutes including *β*-TCP could be effectively used in rapid bone healing after the cyst enucleation.

A randomized controlled trial reported that bone graft does not contribute to increased new bone volume and density between absorbable and non-absorbable membranes [15]. In addition, Dahlin et al. showed that bone density change was not different on the cystic defect between the groups with and without membrane [14]. In this study, the proportion of new bone/remained bone substitutes was not different among the groups at 8 weeks after surgery. However, the experimental group was observed lower proportion of remained bone substitutes, and the proportion was maintained about 20% during postoperative 8 weeks. A proper bone healing outcomes of jaw defect could be achieved if the periosteum was repaired with the barrier membrane [27]. Therefore, the collagen membrane seems to have positive effect of promoting early degradation of the biphasic scaffold by additional mechanical stability. In addition, Kitayama et al. reported that a biphasic scaffold (10% HA and 90% TCP) with a hydroxyapatite-containing collagen membrane and a non-cross-linked collagen membrane was showed superior results, about 35% of new bone formation, compared with deproteinized bovine bone without a membrane [28]. Recently, another biphasic scaffold (20% HA and 80% TCP) with ultraviolet cross-linked membrane was showed high biocompatibility and 36% of new bone formation, comparable with chemically cross-linked collagen membrane [29]. Thus, further research should be conducted to decide an optimal proportion of HA and *β*-TCP in the biphasic scaffold and an effective type of membrane for enhance new bone formation.

A biomechanical role for the membrane has been proposed to stabilizing and protecting the nascent clot with appropriate mechanical properties necessary for osteogenesis [30]. The bone regeneration is influenced by the micro-environment, and the initially formed blood clot may influence the migration of cells and vascularization [31]. However, few studies that demonstrated the processes with the membrane provided evidence that the membrane could act a biological role in bone regeneration with the bone grafts [32]. With regard to our results with membrane, the stability of early formed blood clot could induce early biodegradation of the biphasic bone substitute and new bone formation [33].

After bone graft for the cystic defect, infection was reported 17.6% of thirty-four cases [1]. The infection lead to delay bone healing, and cause of the infection could be related with secondary inflammatory reaction mainly due to wound dehiscence [34]. With considering the membrane coverage, the grafts could endure the infection even if the membrane was exposed after wound dehiscence [35]. In particular, rapidly resorbable membrane could even be effective as barrier layer [36], and the collapse of adjacent soft tissues could be prevented through the membrane [37, 38]. Although we could not reveal the infection-related aspect in this experiment, the biodegradable membrane could be suggested to use in the large defects, which has high risk of wound dehiscence, infection, and recurrence for enhancing stability of grafted materials.

## Conclusions

In canine mandibular defect, the biodegradable polymer membrane contributed to the early biodegradation of *β-*TCP in the biphasic alloplastic bone substitutes. However, the membrane had not affected the bone regeneration capacity, including the proportion of new bone volume and remained bone substitutes.

## Data Availability

The datasets used during the current study are available from the corresponding author on reasonable request.

## Abbreviations

HA: Hydroxyapatite
TCP: Tricalcium phosphate
BV: Newly formed bony volume
RG: Remaining grafting volume

## References

[1] Lim SY, Yeo DS, Lee HJ, Kim HK, An KM, Sohn DS (2006). A clinicostatical study of jaw cyst between 2001~2005. J Korean Assoc Oral Maxillofac Surg.

[2] Chacko R, Kumar S, Arvind PA (2015). Spontaneous bone regeneration after enucleation of large jaw cysts: a digital radiographic analysis of 44 consecutive cases. J Clin Diagn Res.

[3] Ihan Hren N, Miljavec M (2008). Spontaneous bone healing of the large bone defects in the mandible. Int J Oral Maxillofac Surg.

[4] Bertoldi C, Zaffe D, Consolo U (2008). Polylactide/polyglycolide copolymer in bone defect healing in humans. Biomaterials..

[5] Kim YK, Lee JY, Kim SG, Lim SC (2013). Guided bone regeneration using demineralized allogenic bone matrix with calcium sulfate: case series. J Adv Prosthodont.

[6] Mitchell R (1992). An evaluation of bone healing in cavities in the jaws implanted with a collagen matrix. Br J Oral Maxillofac Surg.

[7] Schepers EJ, Ducheyne P (1997). Bioactive glass particles of narrow size range for the treatment of oral bone defects: a 1-24 month experiment with several materials and particle sizes and size ranges. J Oral Rehabil.

[8] Forssell K, Forssell H, Kahnberg KE (1988). Recurrence of keratocysts: a long-term follow-up study. Int J Oral Maxillofac Surg.

[9] Motamedi M, Talesh K (2005). Management of extensive dentigerous cysts*.* British. Dental J.

[10] Huang IY, Lai ST, Chen CH, Chen CM, Wu CW, Shen YH (2007). Surgical management of ameloblastoma in children. Oral Surg Oral Med Oral Pathol Oral Radiol Endod.

[11] Jang K, Lee JH, Oh SH, Ham BD, Chung SM, Lee JK, Ku JK (2020). Bone graft materials for current implant dentistry. J Dental Implant Res.

[12] Gauthier O, Bouler JM, Aguado E, Pilet P, Daculsi G (1998). Macroporous biphasic calcium phosphate ceramics: influence of macropore diameter and macroporosity percentage on bone ingrowth. Biomaterials..

[13] Ettl T, Gosau M, Sader R, Reichert TE (2012). Jaw cysts - filling or no filling after enucleation? A review. J Craniomaxillofac Surg.

[14] Dahlin C, Gottlow J, Linde A, Nyman S (1990). Healing of maxillary and mandibular bone defects using a membrane technique. An experimental study in monkeys. Scand J Plast Reconstr Surg Hand Surg.

[15] Santamaria J, Garcia AM, de Vicente JC, Landa S, Lopez-Arranz JS (1998). Bone regeneration after radicular cyst removal with and without guided bone regeneration. Int J Oral Maxillofac Surg.

[16] Park SA, Lee SH, Kim WD (2011). Fabrication of porous polycaprolactone/hydroxyapatite (PCL/HA) blend scaffolds using a 3D plotting system for bone tissue engineering. Bioprocess Biosyst Eng.

[17] Kim JY, Cho DW (2009). Blended PCL/PLGA scaffold fabrication using multi-head deposition system. Microelectron Eng.

[18] Hjorting-Hansen E, Andreasen JO (1971). Incomplete bone healing of experimental cavities in dog mandibles. Br J Oral Surg.

[19] Barboza EP, Duarte MEL, Geolás L, Sorensen RG, Riedel GE, Wikesjö UME (2000). Ridge augmentation following implantation of recombinant human bone morphogenetic protein-2 in the dog. J Periodontol.

[20] Kim YK, Yun PY, Lim SC, Kim SG, Lee HJ, Ong JL (2008). Clinical evaluations of OSTEON as a new alloplastic material in sinus bone grafting and its effect on bone healing. J Biomed Mater Res B Appl Biomater.

[21] Kondo N, Ogose A, Tokunaga K, Umezu H, Arai K, Kudo N, Hoshino M, Inoue H, Irie H, Kuroda K, Mera H, Endo N (2006). Osteoinduction with highly purified beta-tricalcium phosphate in dog dorsal muscles and the proliferation of osteoclasts before heterotopic bone formation. Biomaterials..

[22] Chazono M, Tanaka T, Komaki H, Fujii K (2004). Bone formation and bioresorption after implantation of injectable beta-tricalcium phosphate granules-hyaluronate complex in rabbit bone defects. J Biomed Mater Res A.

[23] Jensen SS, Broggini N, Hjorting-Hansen E, Schenk R, Buser D (2006). Bone healing and graft resorption of autograft, anorganic bovine bone and beta-tricalcium phosphate. A histologic and histomorphometric study in the mandibles of minipigs. Clin Oral Implants Res.

[24] Jensen SS, Bornstein MM, Dard M, Bosshardt DD, Buser D (2009). Comparative study of biphasic calcium phosphates with different HA/TCP ratios in mandibular bone defects. A long-term histomorphometric study in minipigs. J Biomed Mater Res B Appl Biomater.

[25] Buser D, Hoffmann B, Bernard JP, Lussi A, Mettler D, Schenk RK (1998). Evaluation of filling materials in membrane--protected bone defects. A comparative histomorphometric study in the mandible of miniature pigs. Clin Oral Implants Res.

[26] Horch HH, Sader R, Pautke C, Neff A, Deppe H, Kolk A (2006). Synthetic, pure-phase beta-tricalcium phosphate ceramic granules (Cerasorb<sup>®</sup>) for bone regeneration in the reconstructive surgery of the jaws. Int J Oral Maxillofac Surg.

[27] Zwahlen RA, Cheung LK, Zheng LW, Chow RL, Li T, Schuknecht B, Gratz KW, Weber FE (2009). Comparison of two resorbable membrane systems in bone regeneration after removal of wisdom teeth: a randomized-controlled clinical pilot study. Clin Oral Implants Res.

[28] Kitayama S, Wong LO, Ma L, Hao J, Kasugai S, Lang NP, Mattheos N (2016). Regeneration of rabbit calvarial defects using biphasic calcium phosphate and a strontium hydroxyapatite-containing collagen membrane. Clin Oral Implants Res.

[29] Hong I, Khalid AW, Pae HC, Cha JK, Lee JS, Paik JW, Jung UW, Choi SH (2019). Distinctive bone regeneration of calvarial defects using biphasic calcium phosphate supplemented ultraviolet-crosslinked collagen membrane. J Periodontal Implant Sci.

[30] Hämmerle CH, Schmid J, Lang NP, Olah AJ (1995). Temporal dynamics of healing in rabbit cranial defects using guided bone regeneration. J Oral Maxillofac Surg.

[31] Liu J, Kerns DG (2014). Mechanisms of guided bone regeneration: a review. Open Dent J.

[32] Omar O, Elgali I, Dahlin C, Thomsen P (2019). Barrier membranes: more than the barrier effect?. J Clin Periodontol.

[33] El-Rashidy AA, Roether JA, Harhaus L, Kneser U, Boccaccini AR (2017). Regenerating bone with bioactive glass scaffolds: a review of in vivo studies in bone defect models. Acta Biomater.

[34] Horowitz I, Bodner L (1989). Use of xenograft bone with aspirated bone marrow for treatment of cystic defect of the jaws. Head Neck.

[35] Eskan MA, Girouard ME, Morton D, Greenwell H (2017). The effect of membrane exposure on lateral ridge augmentation: a case-controlled study. Int J Implant Dent.

[36] Kodama T, Minabe M, Hori T, Watanabe Y (1989). The effect of various concentrations of collagen barrier on periodontal wound healing. J Periodontol.

[37] Blecher JC, Lemperle SM, Howaldt HP (2000). Osteoplasty of extensive jaw defects by protected bone regeneration using large pore resorbable implant. Mund Kiefer Gesichtschir.

[38] Ma JL, Pan JL, Tan BS, Cui FZ (2009). Determination of critical size defect of minipig mandible. J Tissue Eng Regen Med.

